# Radiotherapy-synergized in situ hydrogel vaccine with engineered *Lactococcus lactis* FOLactis potentiates anti-tumor immunity in pancreatic cancer

**DOI:** 10.3389/fimmu.2026.1789212

**Published:** 2026-06-29

**Authors:** Jingjing Chen, Qiaoli Wang, Junmeng Zhu, Xinyuan Bai, Haochen Tang, Xiang Kong, Lu Zou, Qinghua Zheng, Yingxin Wang, Yan Zhao, Baorui Liu, Fanyan Meng, Juan Du

**Affiliations:** 1 Department of Oncology, Nanjing Drum Tower Hospital, Clinical College of Nanjing Drum Tower Hospital, Nanjing University of Chinese Medicine, Nanjing, China; 2 The Comprehensive Cancer Center of Nanjing Drum Tower Hospital, Affiliated Hospital of Medical School, Nanjing University, Nanjing, China; 3 Nanjing Drum Tower Hospital, Affiliated Hospital of Medical School, Nanjing University, Nanjing, China; 4 Department of Laboratory Medicine, Nanjing Drum Tower Hospital, Clinical College of Nanjing Medical University, Nanjing, China

**Keywords:** hydrogel, in situ tumor vaccine, *Lactococcus lactis*, pancreatic cancer, radiotherapy

## Abstract

**Background:**

Immunotherapy has emerged as a promising strategy for pancreatic cancer. Radiotherapy not only mediates direct tumor cell killing but also provides a source of tumor antigens for dendritic cells (DCs) uptake and presentation via the induction of immunogenic cell death (ICD). However, the limited antigen presentation efficiency following radiotherapy and the rapid enzymatic degradation of adjuvants within the tumor microenvironment hinder the subsequent efficacy of immunotherapy.

**Methods:**

We developed an *in situ* hydrogel vaccine, Gel-FOLactis. Combined with 8Gy radiotherapy for treatment. This *in situ* vaccine employs a thermosensitive P407 hydrogel to encapsulate engineered *Lactococcus lactis* (FOLactis), enabling sustained intratumoral delivery of the immune-stimulating cytokines Fms-like tyrosine kinase 3 ligand (Flt3L) and co-stimulator OX40 ligand (OX40L).

**Results:**

The sustained release of Flt3L recruits and expands conventional type 1 dendritic cells, enhancing their antigen presentation capability for radiotherapy-released antigens. Concurrently, OX40L promotes the activation of tumor-infiltrating effector T cells. This synergy efficiently initiates a potent antigen-specific immune response, leading to improved tumor eradication.

**Conclusions:**

The combined therapy of RH-FOLactis significantly enhanced the anti-tumor immune response and successfully transformed the immunosuppressive tumor microenvironment in pancreatic cancer from "cold" to "hot". These findings highlight the potential of RH-FOLactis as a novel and effective treatment strategy.

## 1 Introduction

Pancreatic ductal adenocarcinoma (PDAC) remains one of the most lethal malignancies, characterized by a highly immunosuppressive tumor microenvironment (TME) and a dismal 5-year survival rate of approximately 11% (1). The dense desmoplastic stroma, hypoxic niche, dysfunctional and numerically insufficient DCs and abundance of immunosuppressive cells severely limit the infiltration and function of cytotoxic T lymphocytes (CTLs), rendering PDAC largely refractory to conventional immunotherapies, including checkpoint inhibitors (2). Consequently, there is an urgent need for novel strategies capable of effectively reprogramming the “cold” PDAC TME into an immunoreactive “hot” state to unleash potent anti-tumor immunity.

Recently, radiotherapy (RT) represents a standard-of-care modality for PDAC (3). Beyond its direct cytotoxic effects, sub-lethal RT can induce ICD, releasing tumor-associated antigens (TAAs) and damage-associated molecular patterns (DAMPs) (4), and concurrently remodel the TME by enhancing the pro-inflammatory molecules’ release and immune cell infiltration (5–7). This “in situ vaccination” effect can synergize with immunotherapies by enhancing antigen availability and priming tumor-specific T cells (8, 9). However, the ability of RT alone to overcome PDAC immunosuppression is limited. Radiotherapy-induced immune responses are often transient and fail to elicit potent anti-tumor immunity owing to insufficient DCs-mediated antigen presentation (10, 11). Thus, an efficient platform is urgently required to in situ enrich immune adjuvants and recruit DCs to tumors, enhancing antigen presentation and achieving durable immunity.

Leveraging bacteria as drug delivery vehicles is an emerging anti-tumor strategy, demonstrating significant potential in precision therapy and immune activation (12, 13). Certain strains preferentially colonize tumors due to the hypoxic and immunosuppressive TME, and their pathogen-associated molecular patterns (PAMPs) can potently activate innate and adaptive immune responses (14, 15). Critically, this strategy is supported by growing evidence that the microbiome, including the intratumoral microbiota, is a key determinant of cancer treatment response. The microbiota intricately shapes the tumor microenvironment and systemic anti-tumor immunity, with its composition and derived metabolites being pivotal determinants of immunotherapy efficacy (16, 17).​ Thus, the engineered use of bacteria represents a targeted modality within the broader, promising frontier of microbiome modulation for cancer treatment. Our group previously engineered *Lactococcus lactis* expressing a fusion protein of FMS-like tyrosine kinase 3 ligand (Flt3L) and OX40 ligand (OX40L), termed FOLactis. Flt3L promotes DCs recruitment and maturation (18), while OX40L provides a critical co-stimulatory signal for T cell activation and survival (19). We demonstrated that intratumoral injection of FOLactis could remodel the TME, enhance DCs-mediated antigen presentation, increase CTL infiltration, and induce significant tumor regression in colorectal cancer models (20). However, a key limitation for clinical translation is the rapid clearance of free bacteria from the injection site, potentially reducing sustained immune stimulation and therapeutic efficacy.

To achieve spatiotemporally controlled release of vaccine components,​ hydrogel-based delivery systems represent a compelling strategy by providing enhanced localized and sustained release of therapeutic agents​ (21). Recently, hydrogels have emerged as powerful platforms for cancer immunotherapy, enabling the precise delivery and sustained presentation of immunomodulatory agents directly within the tumor microenvironment, thereby potently reshaping local and systemic anti-tumor immunity (22–24). Poloxamer 407 (P407), an FDA-approved thermosensitive triblock copolymer, undergoes rapid sol-to-gel transition at body temperature, forming a biodegradable matrix that can encapsulate bioactive cargo and provide controlled, prolonged release at the target site (25, 26). Its excellent biocompatibility and established use in drug delivery make it an ideal candidate for improving bacterial retention (27–29).

**Scheme 1:**
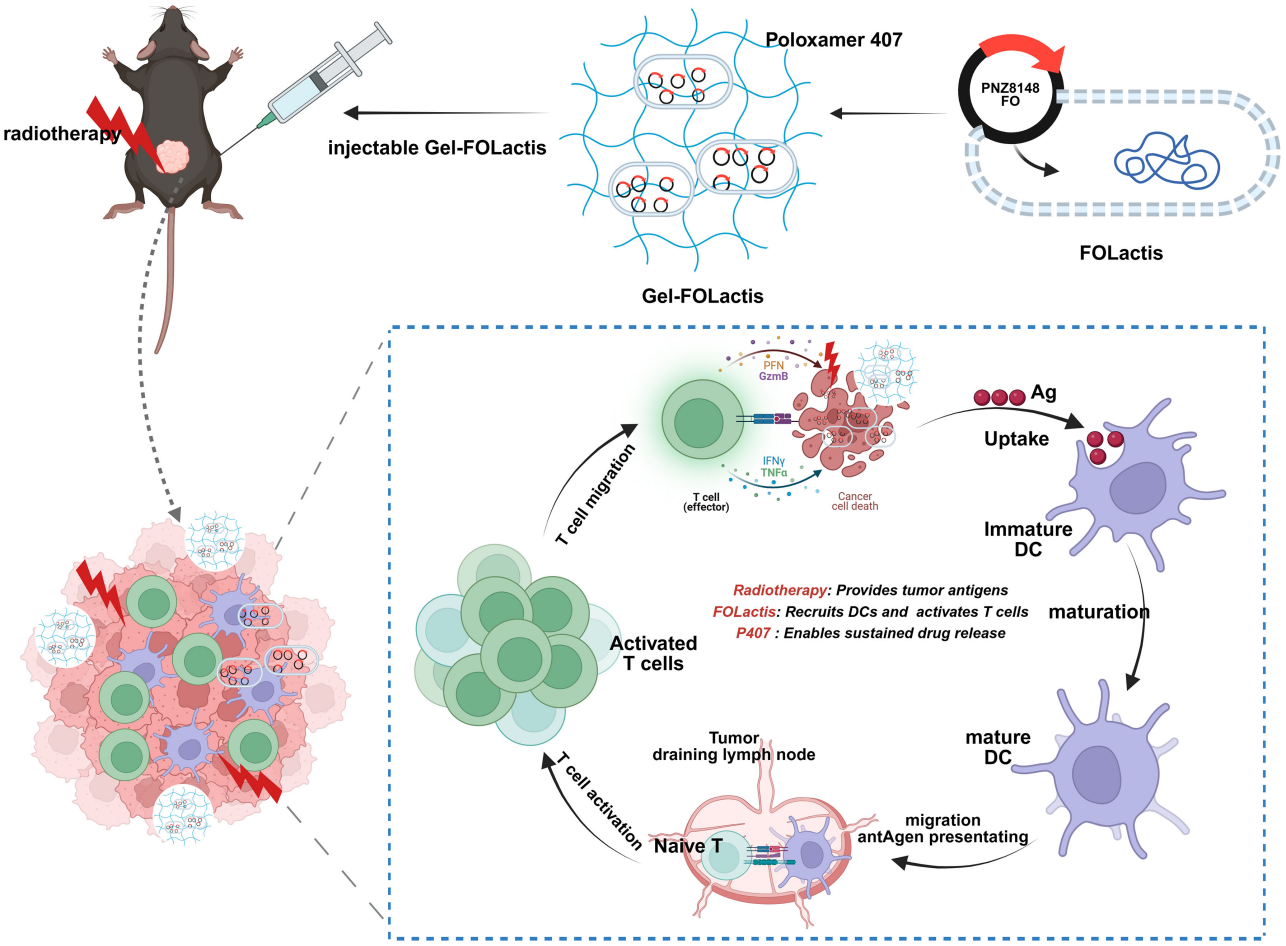
Schematic illustration of the synergistic anti-tumor mechanism of Gel-FOLactis combined with radiotherapy.

In this study, we design a novel combined treatment strategy named RH-FOLactis. This strategy aims to overcome treatment resistance through a triple synergistic mechanism: radiotherapy can kill tumor cells and facilitate the presentation of tumor antigens by DCs; the Flt3L and OX40L secreted by the engineered bacterium FOLactis can respectively promote the recruitment/matured of DCs and the activation/living of T cells, presenting the tumor antigens after radiotherapy, triggering antigen-specific responses, and directly reversing the immunosuppressive microenvironment; while the P407 hydrogel (Gel-FOLactis) achieves the sustained release and retention of FOLactis by encapsulation, solving the clinical translational bottleneck of its rapid clearance in vivo (**Scheme 1**). Studies have shown that Gel-FOLactis can prolong the retention time and maintain biological activity within the tumor. In the syngeneic KPC pancreatic cancer model, we evaluated its anti-tumor efficacy as a single agent and in combination with radiotherapy, and found that the RH-FOLactis combination therapy can synergistically enhance the activation and infiltration of DCs and cytotoxic T lymphocytes, effectively inhibit tumor growth, prolong survival, and without causing significant systemic toxicity. These findings indicate that this study provides a safe and effective new treatment strategy for overcoming pancreatic cancer immunosuppression and lays a solid experimental foundation for clinical translation.

## 2 Results

### 2.1 Characterization of FOLactis-Loaded P407 hydrogels

**Figure 1:**
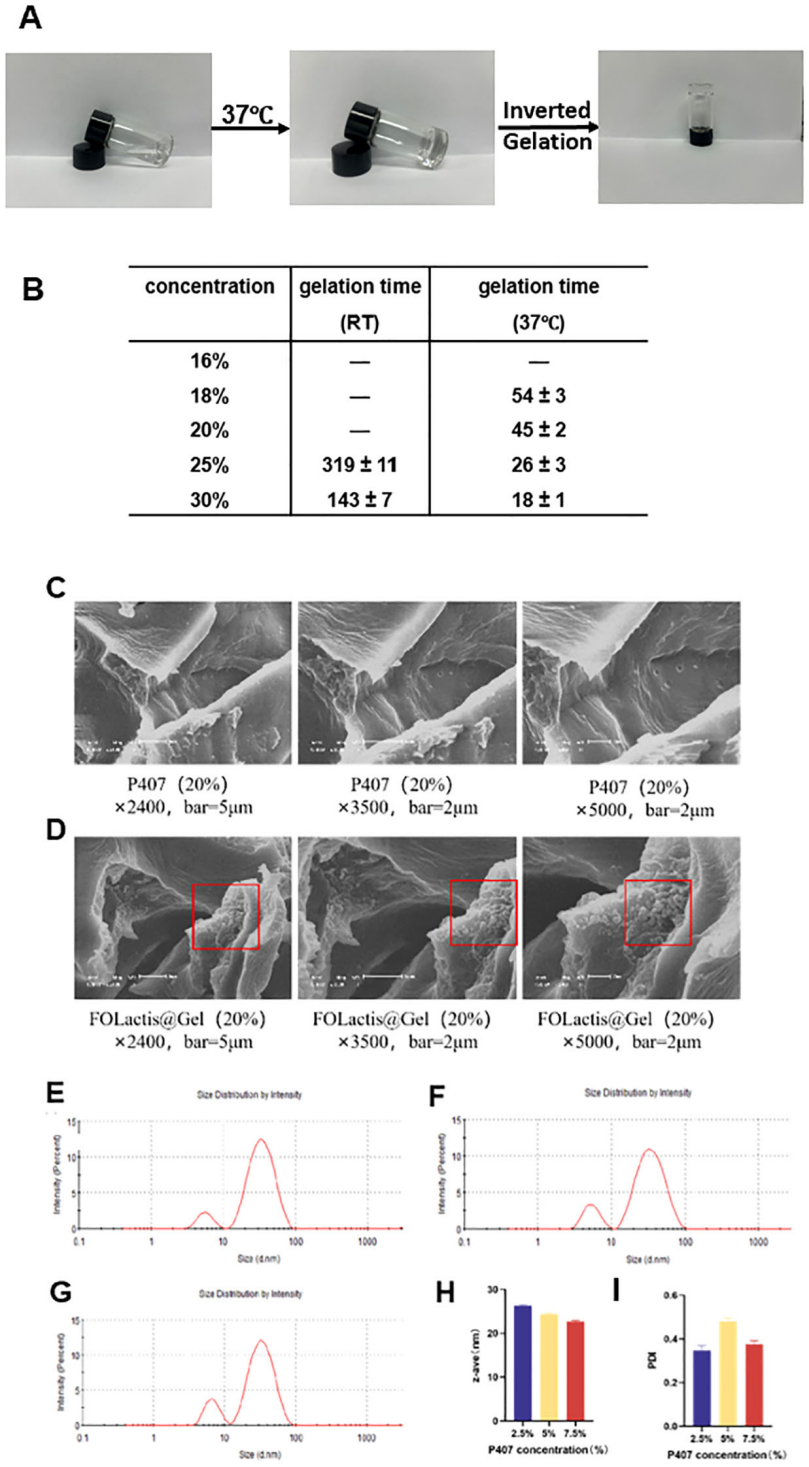
Characterization of thermosensitive P407 hydrogel and FOLactis@Gel formulation​. **(A)** Macroscopic sol-gel transition of 20% P407 hydrogel at 37 °C. **(B)** Gelation time quantification at varying P407 concentrations (16-30%) under room temperature (RT) and physiological temperature (37 °C). Data represent mean ± SEM (n=3). **(C)** Micrographs of blank P407 hydrogel (20%) showing interconnected porous architecture at different magnifications (×2,400, ×3,500, ×5,000). **(D)** FOLactis-loaded hydrogel (FOLactis@Gel) with bacterial aggregates highlighted in red boxes (scale bars: 5μm, 2 μm, 2 μm). **(E-G)** Intensity-weighted size distribution profiles of P407 solutions showing bimodal peaks. **(E)** 2.5% P407​​. **(F)** 5% P407. **(G)** 7.5% P407. **(H, I)** Concentration-dependent effects on **(H)** particle size and **(I)** polydispersity index (PDI) of P407 micelles (2.5-7.5%).

As consistently documented in biomedical engineering literature, the gelation kinetics of poloxamer 407 (P407) hydrogels exhibit temperature- and concentration-dependent behavior. Below the critical gelation temperature (CGT), the system maintains free-flowing properties enabling injectable delivery. Upon thermal elevation beyond the CGT, rapid hydrogel solidification occurs, facilitating localized drug depot formation (**Figure 1A**). To establish an optimal delivery platform, we engineered poloxamer 407 (P407) hydrogels at graded concentrations (16-30%, w/v) and quantified their gelation kinetics under clinically relevant temperatures (**Figure 1B**). At 37 °C (physiological temperature), 16% P407 failed to undergo sol-gel transition, whereas formulations between 18-30% demonstrated progressively accelerated gelation: from 54 ± 3 s (18%) to 18 ± 1 s (30%), confirming concentration-dependent micellar packing. At room temperature-representing injection conditions-critical clinical constraints emerged: ≥25% concentrations solidified prematurely (319 ± 11 s for 25%; 143 ± 7 s for 30%), raising risks of needle clogging during administration, while ≤20% variants retained injectable fluidity indefinitely. This thermoresponsive dichotomy directly informs in vivo functionality: localized depot formation requires rapid gelation (<60 s at 37 °C) to prevent payload diffusion, coupled with sustained RT fluidity (>300 s) to ensure safe injection. The 20% P407 formulation uniquely satisfied both criteria - maintaining free-flowing liquid properties at RT with negligible viscosity change over 10 min (injection window), while transitioning to solid gels within 45 ± 2 s at 37°. This concentration was consequently selected for subsequent therapeutic development as it optimally balances clinical handling safety and localized drug retention.

Cross-sectional SEM analysis of 20% P407 hydrogels revealed an interconnected macroporous network across multiple magnifications (×2400-5000; **Figure 1C**). These channel-like structures (5-20 μm diameter) arise from the ​​thermally induced micellar packing​​ during sol-gel transition, where micellar clusters undergo spontaneous reorganization into ordered lattices upon 37 °C exposure. Crucially, FOLactis-loaded hydrogels demonstrated ​​successful bacterial encapsulation within micellar compartments​​, with the bacteria uniformly distributed throughout the matrix (**Figure 1D**, red arrows). The persistence of porous pathways following encapsulation confirms a dual-function design: (1) Structural integrity maintains physical entrapment of bacteria, while (2) continuous aqueous channels facilitate sustained release during hydrogel dissolution. This architecture permits time-dependent diffusion of FOLactis through the pores, directly enabling controlled payload elution without burst release—a critical feature for in situ vaccine applications.

Dynamic light scattering (DLS) characterization revealed the micellar organization of P407 hydrogels at varying concentrations (2.5%, 5%, and 7.5%). Bimodal size distributions were observed across all formulations (**Figures 1E–G**), with distinct peaks at 5 nm (individual micelles) and 50 nm (micellar aggregates), confirming heterogeneous colloidal solutions. Notably, increasing P407 concentration correlated with reduced average micelle sizes and moderate polydispersity (**Figures 1H, I**).

### 2.2 Sustained release and anti-tumor efficacy of FOLactis-Loaded 20% P407 hydrogel in KPC pancreatic cancer models

**Figure 2:**
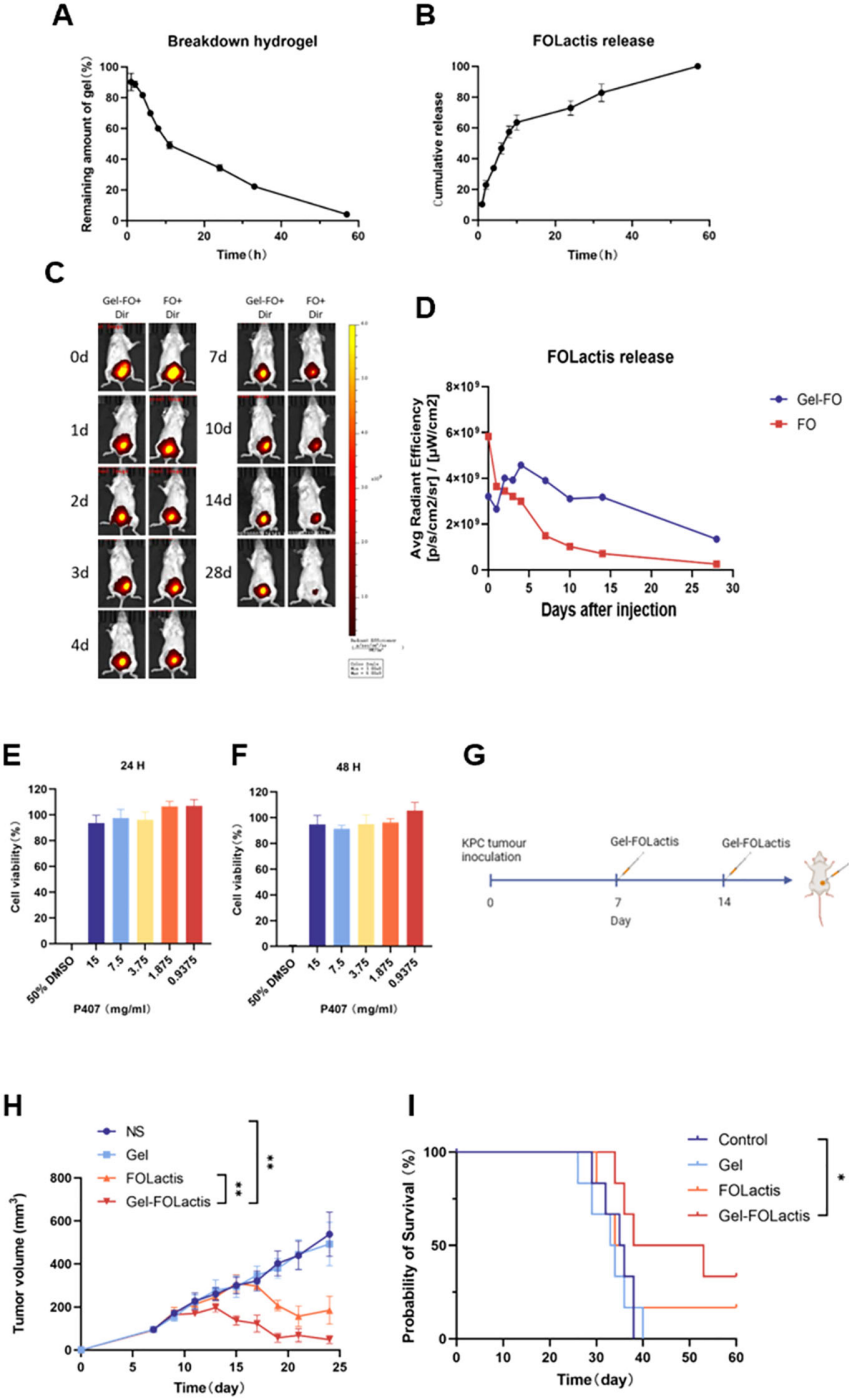
Sustained release and biocompatibility of FOLactis-Loaded 20% P407 Hydrogel: in vitro/in vivo characterization​. **(A)** In vitro degradation kinetics of 20% P407 hydrogel over 60 hours (n=3, mean ± SEM). **(B)** Biphasic FOLactis release profile. burst release within 10 h followed by sustained release (n=3, mean ± SEM). **(C)** In vivo fluorescence imaging comparing Gel-FOLactis and free FOLactis retention in tumors (0–28 days; color scale: radiant efficiency, p/s/cm²/sr). **(D)** Quantified retention curves (Gel-FOLactis vs FOLactis). **(E, F)** Cytocompatibility assessment at 24/48 h (0.9375–15 mg/mL). **(G-I)** P407 hydrogel potentiates FOLactis therapy. **(G)** Study design. **(H)** Average tumor-growth curves of C57BL/6 mice bearing KPC tumor with different treatments as indicated. The mice were peritumorally injected with NS, 20%P407, 5 × 10^9^ FOLactis or 20% P407 + 5 × 10^9^ FOLactis, which were dissolved to a final volume of 100 μl per dose when tumor volume reached about 100 mm^3^. Tumor size was measured every 2–3 days for 25 days. The error bars represented mean ± SEM (n=6). p-values were calculated by two-way ANOVA and Tukey post-test and correction. ns represented p > 0.05, ** represented p <0.01. **(I)** Survival curves of different groups for 60 days. The error bars represented mean ± SEM (n=6). *p*-values were calculated by log-rank (Mantel–Cox) test, * represented *p* < 0.05.

**Figure 2A** demonstrates the degradation profile of the hydrogel. Approximately 50% of the hydrogel was hydrolyzed within 10 hours, while the remaining P407 underwent complete hydrolysis by 60 hours. Concurrently, about 60% of the initial FOLactis payload was released within the first 10 hours, with the remaining 40% gradually released over 60 hours (**Figure 2B**). The bacteria are released concomitant with the degradation of the hydrogel. However, within 10 hours, the release rate of the bacteria is slightly higher than the degradation rate of the hydrogel. The diffusion effect is independent of matrix erosion, indicating that the release of the bacteria is not only affected by the degradation of the gel but also related to its own movement and other factors.

Near-infrared imaging with DIR labeling demonstrated significantly prolonged retention of FOLactis when delivered via P407 hydrogel (**Figures 2C, D**). While free FOLactis exhibited rapid signal attenuation with complete clearance by day 28, Gel-FOLactis demonstrated higher peak radiant efficiency at day 0 and sustained signal persistence through day 28, confirming hydrogel-mediated prolonged retention.

To evaluate the potential cytotoxicity of P407 hydrogel, KPC cells were treated with varying concentrations (0.9375–15 mg/mL) for 24 and 48 hours, followed by CCK-8 assay. As shown in (**Figures 2E, F**), P407 hydrogel did not significantly affect cell viability at any concentration tested, with viability consistently maintained above 80% at both time points. Notably, no concentration- or time-dependent cytotoxic effects were observed, confirming the excellent biocompatibility of P407 hydrogel. These findings align with previous reports demonstrating the safety of polyethylene oxide-based hydrogels as drug/cell carriers.

​Leveraging the prolonged intratumoral retention of Gel-FOLactis (**Figure 2D**) and its established biosafety (**Figures 2E, F**), we assessed its anti-tumor effects in C57BL/6 mice bearing subcutaneous KPC tumors. Tumor cells were subcutaneously inoculated into the left flank, and when the average tumor volume reached ~100 mm³, mice were randomized into four treatment groups: (1) ​​NS​​ (normal saline), (2) ​​Gel​​ (20% P407 hydrogel alone), (3) ​​FOLactis​​ (5×10^9^ CFU free bacteria), and (4) ​​Gel-FOLactis​​ (20% P407 hydrogel + 5×10^9^ CFU FOLactis). All treatments were administered peritumorally. Tumor dimensions and body weight were recorded every 2–3 days to calculate tumor volume (**Figure 2G**). The treatment timeline (**Figure 2G**) was designed to align with the hydrogel’s degradation kinetics (**Figure 2D**), with Gel-FOLactis administered on days 7 and 14 post-tumor inoculation to coincide with (i) peak bacterial viability (confirmed by DIR imaging in **Figures 2C, D**) and (ii) the active tumor stroma formation phase, thereby maximizing immunomodulatory effects while leveraging the hydrogel’s sustained retention properties.​ As shown in **Figure 2H**, tumor growth curves revealed that ​​Gel-FOLactis​​ significantly suppressed tumor progression after the first administration, whereas tumors in other groups continued to grow. Following the second dose, both ​​FOLactis​​ and ​​Gel-FOLactis​​ exhibited marked anti-tumor effects, with ​​Gel-FOLactis​​ inducing complete regression (CR) in ​​33.3% (2/6)​​ of mice (**Figure 2I**). In contrast, tumors in ​​NS​​ and ​​Gel​​ groups progressed unabated.

### 2.3 Synergistic anti-tumor efficacy and safety profile of Gel-FOLactis combined with radiotherapy in pancreatic cancer

In the field of oncology research, although peritumoral injection of Gel-FOLactis induced tumor regression, some tumors continued to progress slowly, indicating the presence of immune escape mechanisms within the tumor microenvironment. Radiotherapy, as a well-established cancer treatment modality, can expose calreticulin (CRT) on the surface of tumor cells (**Supplementary Figure 8**, Supporting Information), thereby inducing immunogenic cell death (ICD) and releasing tumor-associated antigens for uptake and presentation by dendritic cells. It also modulates the tumor microenvironment, enhancing antigen-specific immune responses and ultimately reprogramming the immunosuppressive microenvironment. Therefore, combining radiotherapy with immunotherapy was proposed to enhance the therapeutic efficacy of immunotherapy.

**Figure 3:**
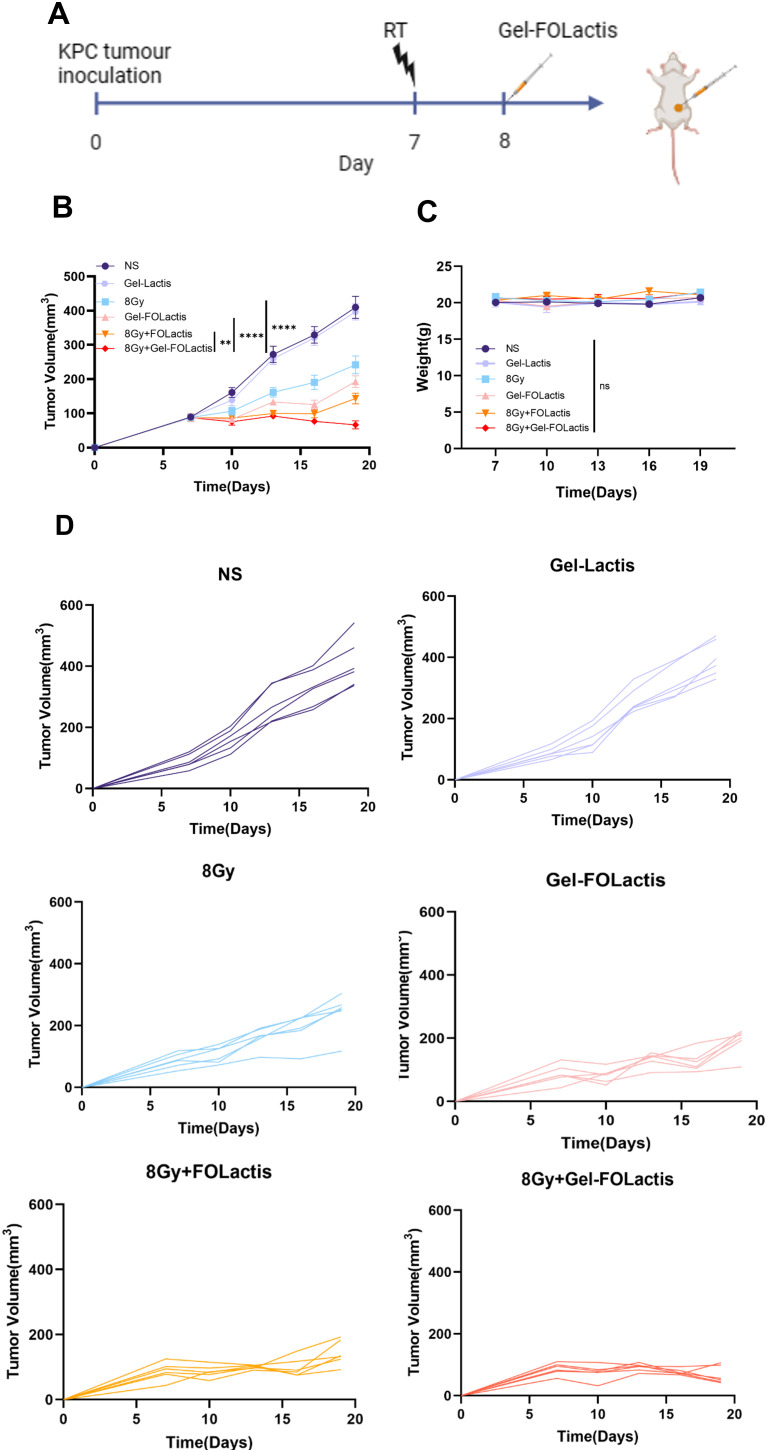
Synergistic antitumor effects of Gel-FOLactis and radiotherapy in KPC pancreatic cancer models. **(A)** Schematic timeline of combined therapy: KPC tumor cells were inoculated subcutaneously in C57BL/6 mice. Radiotherapy (RT, 8Gy) was administered on Day 7, followed by peritumoral injections of Gel-FOLactis (20% P407 + 5×10^9^ CFU FOLactis) on Day 8. **(B)** Average tumor growth curves of C57BL/6 mice bearing KPC tumors under different treatments: NS, Gel-Lactis, RT alone, Gel-FOLactis, 8Gy+FOLactis, and 8Gy+Gel-FOLactis. Tumor size was measured every 2–3 days. The error bars represented mean ± SEM (n=6). p-values were calculated by two-way ANOVA and Tukey post-test and correction. ns represented p > 0.05, * represented p<0.05, ** represented p<0.01, *** represented p<0.001 and **** represented p<0.0001. **(C)** Body weight changes of mice in different groups, with error bars representing mean ± SEM (n=6). p-values were calculated using unpaired Student’s t-test. **(D)** Individual tumor growth trajectories for each mouse in the six main treatment groups.

The experimental treatments were categorized into six groups according to the different therapeutic interventions, as illustrated in **Figure 3A**. KPC tumor cells were inoculated on Day 0. Radiotherapy (8 Gy) was administered on Day 7, and the first dose of Gel-FOLactis was given on Day 8. The 8 Gy radiation dose was selected based on its established efficacy in inducing immunogenic cell death (ICD), a process that releases tumor-associated antigens and danger signals, thereby priming an adaptive immune response. This dose is well-supported in the radiobiology literature as optimal for balancing tumor ablation with immune activation in the context of combination immunotherapy (8, 30). As shown in the figure, tumor growth kinetics revealed that the combination treatment of 8Gy radiotherapy and Gel-FOLactis was significantly more effective than either monotherapy or the control. Specifically, the 8Gy+Gel-FOLactis group achieved the most robust tumor control, with tumor volumes markedly reduced compared to the NS group and Gel-Lactis group (**Figure 3B**). The individual volume curves of the groups (**Figure 3D**) further verified this.

**Figure 4:**
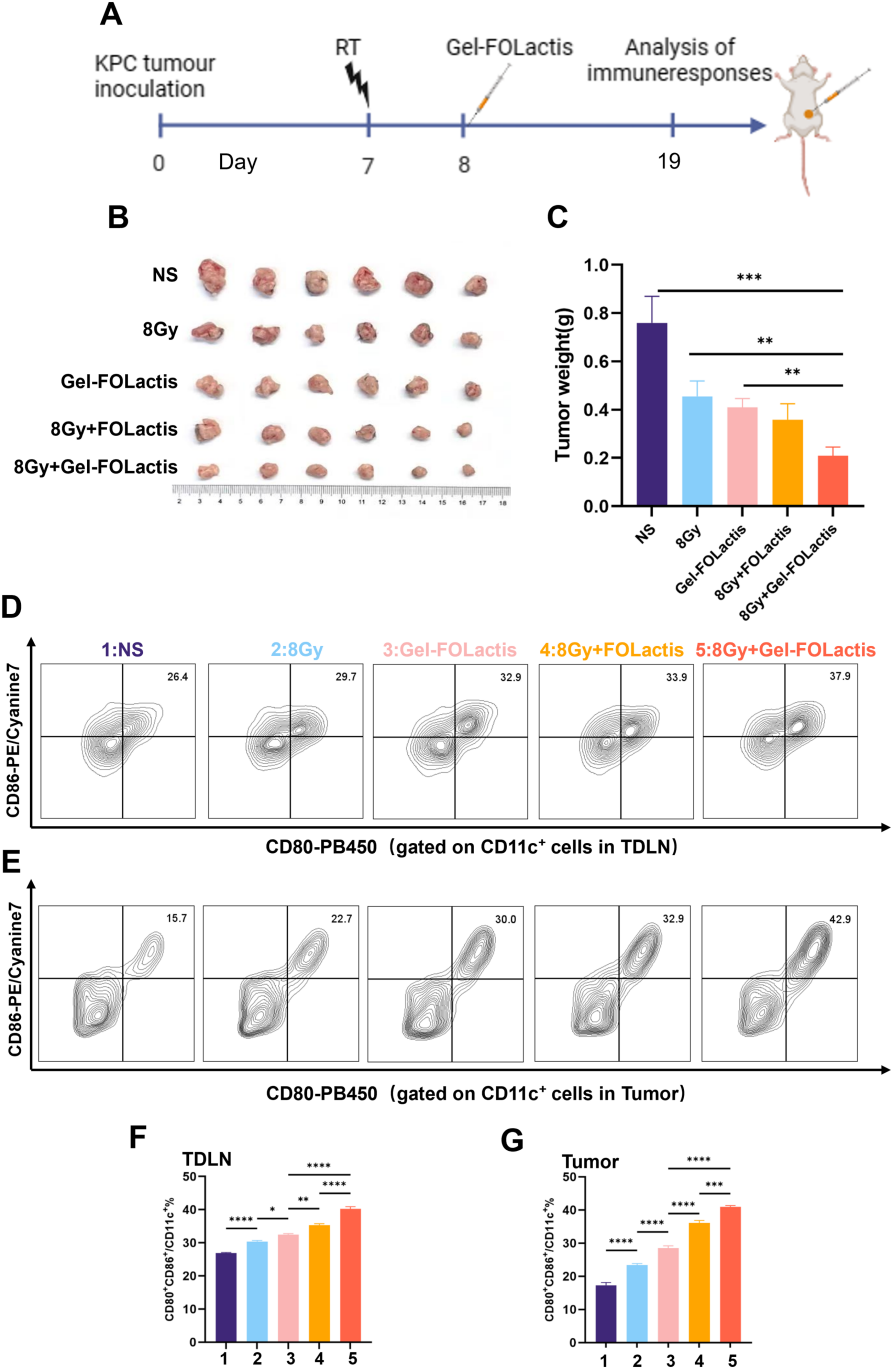
Research on the anti-tumor mechanism of Gel-FOLactis combined with radiotherapy (RT). **(A)** Schematic timeline of combined therapy: KPC tumor cells were inoculated subcutaneously in C57BL/6 mice. Radiotherapy (RT, 8Gy) was administered on Day 7, followed by peritumoral injections of Gel-FOLactis (20% P407 + 5×10^9^ CFU FOLactis) on Day 8. Tumor analysis and immune response evaluation were conducted on Day 19. **(B)** Representative images of excised tumors from each treatment group at the endpoint (Day 19). **(C)** Quantification of tumor weights at the endpoint (Day 19). Data represent mean ± SEM (n=6). *p*-values were determined by one-way ANOVA with Tukey’s *post-hoc* test. **(D)** Representative flowchart of CD80^+^CD86^+^ cells in lymph nodes in each group. **(E)** Representative flowchart of CD80^+^CD86^+^ cells in tumor tissues in each group. **(F)** The proportion of CD80^+^CD86^+^ cells in each group of lymph nodes. **(G)** The proportion of CD80^+^CD86^+^ cells in each group of tumors (mean ± SEM, n=6; ​ ns represented *p* > 0.05, * represented *p* <0.05, ** represented *p*<0.01, *** represented *p*<0.001 and **** represented *p*<0.0001).

The tumor weight in the 8Gy+Gel-FOLactis group was significantly lower than that in the NS group, the 8Gy group, and the Gel-FOLactis group, confirming that the combined treatment had the most effective inhibitory effect on the tumor (**Figure 4C**).The macroscopic photos of the tumor morphology (**Figure 4B**) clearly show that the tumor volume in the NS group was large; the tumor volume in the 8Gy group decreased but did not completely disappear; the tumor in the Gel-FOLactis group was significantly smaller than that in the NS group; most tumors in the 8Gy+Gel-FOLactis group almost completely disappeared, with only a very small amount of tissue remaining, and the morphological differences were consistent with the volume/weight data. Taken together, these findings demonstrate a synergistic effect between Gel-FOLactis and radiotherapy, suggesting that radiotherapy can enhance the immunotherapeutic activity of Gel-FOLactis.

To evaluate the safety profile of the combined treatments in a subcutaneous KPC pancreatic cancer xenograft model, we monitored the body weight changes of mice throughout the treatment period. During the treatment, the body weights across all experimental groups remained relatively stable, with no statistically significant differences observed (**Figure 3C**). Additionally, major organ tissues (heart, liver, spleen, lung, and kidney) were collected from tumor-bearing mice in each treatment group for histopathological analysis via H&E staining (**Supplementary Figure 1**, Supporting Information). The results revealed no evident pathological alterations in these organs, further supporting the safety of combining Gel-FOLactis with radiotherapy in the treatment of KPC pancreatic cancer in this murine model.

To further evaluate the bacterial distribution and colonization characteristics of Gel-FOLactis in the body, we conducted colony count analyses on the tumors, tumor-draining lymph nodes (TDLNs), distant organs (heart, liver, spleen, lungs, kidneys), and blood of the mice in each group on the 4th day after treatment (**Supplementary Figure 3**, Supplementary Information). The results showed that in the 8Gy+Gel-FOLactis group, the bacterial load in the tumor tissue was higher than that in other tissues, suggesting that Gel-FOLactis could effectively colonize and accumulate in the tumor microenvironment. while the bacterial load in the tumors of the 8Gy+FOLactis group was lower than that of the 8Gy+Gel-FOLactis group. In the tumor-draining lymph nodes, the bacterial load in the 8Gy+Gel-FOLactis group was slightly higher than that in the 8Gy+FOLactis group, indicating the migration of bacteria to the local lymph nodes. In the distant organs and blood, no high-load bacteria were detected in both groups, suggesting that combined radiotherapy could significantly enhance the tumor-targeting ability of Gel-FOLactis and reduce the risk of systemic spread. This result provides direct evidence for the tumor-selective effect of Gel-FOLactis combined with radiotherapy and further supports the safety basis for its clinical translation.

### 2.4 Mechanistic insights into the synergistic anti-tumor effects of Gel-FOLactis combined with radiotherapy in pancreatic cancer

To further understand the immune-related mechanisms underlying its synergistic anti-tumor effect, on the 15th day after the last treatment, tumor and draining lymph node tissues from the mice were collected, and the immune-related cells within them were analyzed (**Figure 4A**).The results showed that the numbers of CD80^+^ and CD86^+^ cells in the tumors and draining lymph nodes of the 8Gy, Gel-FOLactis, 8Gy+FOLactis, and 8Gy+Gel-FOLactis groups all increased (**Supplementary Figures 2A–D**, Supporting Information). After combined therapy, the number of mature DCs (CD11c^+^CD80^+^CD86^+^) in the tumors and draining lymph nodes significantly increased (**Figures 4D–G**; **Supplementary Figure 5**, Supporting Information). To verify whether the encapsulation of bacteria within the hydrogel affects the biological activity of the released Flt3L and OX40L, we incubated the hydrogel, FOLactis, or Gel-FOLactis with BMDCs in vitro for 24 hours. Result showed that compared with the blank Gel control group, the maturation markers of dendritic cells (CD80 and CD86) in the FOLactis group and Gel-FOLactis group were significantly upregulated (**Supplementary Figure 7**, Supporting Information). Importantly, the maturation levels between the Gel-FOLactis group and the unencapsulated FOLactis group were comparable, which confirmed that the encapsulation of the hydrogel could maintain the functional integrity of the immune stimulating proteins.

**Figure 5:**
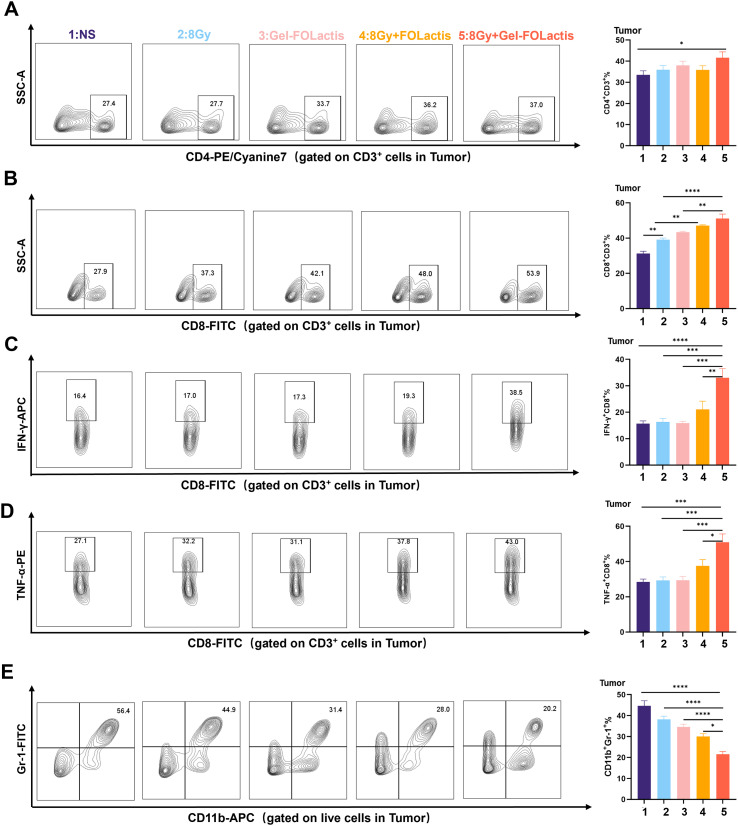
Research on the anti-tumor mechanism of Gel-FOLactis combined with radiotherapy (RT). **(A)** Representative flowchart of CD4^+^ T cells in tumor tissues and the proportion of CD3^+^CD4^+^ cells in each group. **(B)** Representative flowchart of CD8^+^ T cells in tumor tissues and the proportion of CD3^+^CD8^+^ cells in each group. **(C)** Representative flowchart of CD8^+^IFN-γ^+^ cells in tumor tissues and the proportion of CD8^+^IFN-γ^+^ cells in each group. **(D)** Representative flowchart of CD8^+^TNF-α^+^ cells in tumor tissues and the proportion of CD8^+^TNF-α^+^ cells in each group. **(E)** Representative flowchart of CD11b^+^Gr-1^+^ cells in tumor tissues and the proportion of CD11b^+^Gr-1^+^ cells in each group (mean ± SEM, n=6; ns represented p>0.05, *represented p<0.05, **represented p<0.01, ***represented p<0.001 and ****represented p<0.0001).

To assess the infiltration level of T cells in tumor tissues, we analyzed the proportions of CD4^+^ (**Figure 5A**) and CD8^+^ (**Figure 5B**) T cells in CD3^+^T cells (**Supplementary Figure 4**, Supporting Information). The results indicated that the 8Gy+Gel-FOLactis group could promote the infiltration of CD4^+^T cells within the tumor, providing a synergistic signal for anti-tumor immunity. The proportion of CD8^+^T cells also significantly increased in the 8Gy+Gel-FOLactis group, suggesting that the combined treatment could enhance the recruitment and residence of tumor-specific CD8^+^T cells. Furthermore, to investigate the modulation of immunosuppressive cells within the tumor microenvironment, we analyzed the frequency of myeloid-derived suppressor cells (MDSCs, Gr-1^+^CD11b^+^) (**Figure 5E**, **Supplementary Figure 6**, Supporting Information). Compared to the control and monotherapy groups, the 8Gy+Gel-FOLactis group exhibited a significant reduction in the proportion of MDSCs. This decrease in immunosuppressive cells suggests that the combined treatment effectively alleviates the local immune suppression, thereby facilitating the anti-tumor immune response. To evaluate the cytotoxic function of CD8^+^ T cells, we detected the expression of IFN-γ (**Figure 5C**) and TNF-α (**Figure 5D**) in tumor-infiltrating CD8^+^T cells. The proportion of CD8^+^IFN-γ^+^T cells in the 8Gy+Gel-FOLactis group was significantly higher than that in other groups, indicating that the combined treatment could significantly enhance the effector function of CD8^+^T cells within the tumor and improve their cytotoxic potential. The proportion of TNF-α^+^ in tumor-infiltrating CD8^+^T cells was also significantly increased in the 8Gy+Gel-FOLactis group, further confirming that the combined treatment could activate the direct cytotoxicity and pro-inflammatory factor release of CD8^+^T cells, enhancing the anti-tumor effect. These results indicate that Gel-FOLactis combined with radiotherapy can reshape the tumor immune microenvironment, activate the anti-tumor immune response, and provide direct evidence for the synergistic mechanism of the combined treatment.

## 3 Materials and methods

### 3.1 Materials

The reagents used in this study were as follows:Poloxamer 407 (P407) purchased from BASF (Germany); M17 Broth purchased from OXOID (UK); Nisin purchased from Sigma-Aldrich (USA); Glucose purchased from Sinopharm Chemical Reagent Co., Ltd. (China); Chloramphenicol purchased from Sangon Biotech (Shanghai, China); DMEM high-glucose medium purchased from Yuanpei Biotechnology (Shanghai, China); Trypsin cell digestion solution purchased from Beyotime Biotechnology (Shanghai, China); Australian fetal bovine serum (FBS) purchased from Gibco (USA); CCK-8 assay kit purchased from Biosharp Biotechnology (China); Mouse antibodies including CD3, CD4, CD8a, CD11c, CD80, CD86, CD11b and Gr-1 all purchased from BioLegend (USA).

#### 3.1.1 Cells

The mouse pancreatic cancer cell line KPC used in the experiment was preserved by the Tumor Center of the Affiliated Drum Tower Hospital of Nanjing University Medical School. KPC The cell lines were cultured in a DMEM high-glucose medium containing 10% bovine serum, 100 U/mL penicillin and 100 μg/mL streptomycin. The culture conditions were: 37°C, 5% CO2.

#### 3.1.2 Animals

In this experiment, SPF-grade C57BL/6 female mice with a body weight of 18–20 g and a gestational age of 5–6 weeks (from Jiangsu Jishu Pharmacogenomics Biotechnology Co., Ltd., Nanjing) were used. They were housed in the SPF-level barrier system of the Experimental Animal Center of Nanjing University School of Medicine, Nanjing. The temperature (20-26 °C), humidity (30-70%), light/dark cycle (between 6 a.m. and 6 p.m.) and food and water were freely accessible. All experimental animal use and operations were approved by the Animal Experiment Management Committee of Nanjing University School of Medicine, Nanjing Hospital of Affiliated Drum Tower (2024AE01111).

### 3.2 Preparation and characterization of Gel-FOLactis

#### 3.2.1 Preparation of Gel-FOLactis

Firstly, prepare the M17 medium. Dissolve 42.3 g/L of M17 powder and 0.5 g of glucose separately in 50 mL of deionized water. Subsequently, sterilize these two solutions at 121 °C under high pressure for 20 minutes. After sterilization, mix them thoroughly. Then, add chloramphenicol to achieve a final concentration of 10 μg/mL. Secondly, perform bacterial cultivation. Inoculate glycerol bacteria onto the M17 medium at a ratio of 1:200. Incubate the inoculated medium at 30 °C for 12–16 hours. After that, transfer the culture to fresh medium at a ratio of 1:100. Monitor the optical density at 600 nm (OD600) until it reaches a value between 0.3 and 0.7. At this point, add the nisin inducer to a final concentration of 10 ng/mL. Continue the induction process for 3–4 hours. Finally, collect the bacteria by centrifugation at 5000 revolutions per minute (rpm) for 20 minutes at 4 °C. After centrifugation, wash the collected bacteria 5 times with sterile physiological saline (NS). Pre-cooled phosphate-buffered saline (PBS, pH = 7.4) was slowly added to the P407 powder. The mixture was then subjected to magnetic stirring at 4 °C overnight to ensure complete dissolution, resulting in a transparent gel solution. Subsequently, the washed FOLactis bacteria were suspended in a 20% P407 hydrogel. The final concentration was adjusted to 5×10^9^ colony-forming units per milliliter (CFU/mL). The suspension was vortexed thoroughly and then allowed to stand at 4 °C for curing and storage.

#### 3.2.2 Characterization of P407

The gelation time was determined via the repeated aspiration method. Specifically, 1 mL of the hydrogel was placed in a water bath maintained at 37 °C. Subsequently, a 250 μL pipette was used to repeatedly aspirate the hydrogel until the pipette tip became blocked. The time at this point was then accurately recorded. The particle size and dispersion index were detected by dynamic light scattering technique (a 2.5%-7.5% gradient concentration P407 solution was prepared and the size of micelles and PDI were measured on the machine, n=3). Using a scanning electron microscope (SEM) to observe the morphology (the samples were frozen at -80°C for 24 hours, dried for 24 hours, loaded onto an aluminum column and then sputtered with gold for plating). The cytotoxicity of P407 hydrogel against KPC pancreatic cancer cells was assessed using the CCK-8 assay. Specifically, logarithmically growing KPC cells were trypsinized, counted, and seeded into 96-well plates at 3,000 cells per well (including experimental, control, and blank wells), followed by pre-culture for 24 h under 37 °C and 5% CO_2_ conditions. Gradient concentrations of P407 hydrogel (0.9375–15 mg/mL) or 50% DMSO (control) were added to respective wells, with the total volume adjusted to 200 μL per well using complete culture medium. After incubation for 24 h or 48 h, the medium was replaced with fresh medium supplemented with 10 μL CCK-8 solution per well, and plates were further incubated for 1–4 h. Absorbance was measured at 450 nm using a microplate reader. Cell survival rates were calculated using the formula: ​​Survival Rate (%) = [(As – Ab)/(Ac – Ab)] × 100%​​, where ​​As​​, ​​Ac​​, and ​​Ab​​ represent the absorbance values of experimental wells (cells + medium + CCK-8 + drug), control wells (cells + medium + CCK-8 without drug), and blank wells (medium + CCK-8 without cells or drug), respectively. The concentration gradient and dual time-point design comprehensively evaluated the biocompatibility of P407 hydrogel.

#### 3.2.3 The in vivo and in vitro release of Gel-FOLactis

The in vitro degradation characteristics of P407 hydrogel were analyzed using the weight difference calculation method (1 mL of hydrogel loaded with FOLactis was gelated at 37°C, and PBS was replaced at regular intervals for weighing to calculate the degradation rate). All experiments were repeated three times, and the temperature of PBS in the degradation experiments was strictly maintained at 37°C to simulate physiological conditions. The release kinetics of FOLactis in the hydrogel were investigated using an in vitro dynamic drug delivery system. 1 mL of 20% P407 hydrogel loaded with FOLactis was added to a 15 mL centrifuge tube and left to gel at 37 °C for 1 minute. Then, 4 mL of pre-warmed PBS solution (at 37 °C) was slowly added along the wall of the tube (to avoid disrupting the gel structure). At the predetermined time points (0, 4, 8, 12, 24, 36, 48, 60 hours), 1 mL of the supernatant was withdrawn and immediately replenished with an equal volume of fresh 37 °C PBS. The OD600 value of each time point sample was detected using a UV spectrophotometer, and the cumulative release amount of FOLactis was calculated using the standard curve (OD600 - CFU). The in vivo release kinetics of FOLactis in the hydrogel were tracked using near-infrared fluorescence in vivo imaging technology. Firstly, the FOLactis cells were collected by centrifugation (5000 rpm, 10 min, 4 °C), washed with PBS, and resuspended in 1 mL of DIR staining solution (10 mM) at room temperature in the dark for 30 minutes. After centrifugation and removal of the supernatant, the free dye was thoroughly removed by washing twice with PBS. The DIR-labeled FOLactis was then loaded onto the P407 hydrogel and injected subcutaneously into C57BL/6 female mice. Fluorescence signal distribution was dynamically monitored using the IVIS Lumina LT small animal in vivo imaging system (excitation/emission wavelengths: 745/800 nm) on days 1, 2, 3, 4, 7, 10, 14, and 28 after injection. The fluorescence intensity of the target area was quantitatively analyzed using the Living Image software.

### 3.3 ICD analysis

KPC cells were cultured in 24-well plates (3×10^4^ cells per well). After 24 hours of culture, the cells were treated with NS or 8Gy for 48 hours, and then the suspended cells and adherent cells were collected. The cells were labeled with anti-CRT primary antibody (1:50) and Alexa Fluor 647-labeled secondary antibody (1:50) respectively, and then detected by flow cytometry.

### 3.4 In vitro BMDC stimulation

BMDCs were differentiated for 6 days. On day 6, BMDCs were seeded in 96-well plates (2×10^5^ cells well^−1^) for 2 days and then co-cultured with Gel, FOLactis (2×10^6^ CFU 100 µL^−1^) and Gel-FOLactis (2×10^6^ CFU 100 µL^−1^) for 48 hours. Subsequently, BMDCs were stained with anti-CD11c, anti-CD80 and anti-CD86 and evaluated by flow cytometry.

### 3.5 In vivo experiments

#### 3.5.1 The efficacy of Gel-FOLactis combined with radiotherapy in the subcutaneous tumor model of pancreatic cancer

Tumor model establishment: Select log-phase KPC pancreatic cancer cells, prepare a single-cell suspension (1×10^7^ cells/mL), and inoculate 100 μL (containing 1×10^6^ cells) into the left inguinal region of a C57BL/6 female mouse. Measure the tumor diameter and the mouse’s weight every 2–3 days starting from the day of inoculation. Grouping and administration: On the 7th day after tumor formation (tumor volume ≈ 100 mm³), randomly group the mice. Prepare Gel-FOLactis and store it on ice, and inject the drug (100 μL per mouse) around the tumor on both sides of the tumor (according to the group). Radiotherapy protocol: Anesthetize the mice by intraperitoneal injection of 0.15 mL 5% chloral hydrate. Cover the non-target tissues with lead plates, and irradiate with a 6 MeV electron beam (source-tumor distance 100 cm, dose rate 250 cGy/min) for 8Gy in a single session. Efficacy monitoring: Measure the tumor length (a) and the maximum cross-sectional diameter (b) every 2–3 days, calculate the volume using the formula (a/2 × b²), and draw the tumor inhibition curve; simultaneously record the weight, behavioral indicators (feeding, activity), and survival time. When the tumor volume exceeds 1500 mm³, implement humane endpoints. Safety evaluation: After the treatment, fix the heart, liver, spleen, lung, and kidney in 4% paraformaldehyde, dehydrate, clear, wax-bond, and section. Perform HE staining and observe the histological changes of the tissues using an optical microscope to evaluate the safety of the treatment. Bacterial colonization in vivo: Bacterial colonization was used to calculate the distribution of bacteria ulteriorly. We harvested tumors, TDLNs, blood, and major organs at indicated times after peritumoral injection of FOLactis or FOLactis/P407. The tissues were then weighed and grinded in NS at 4 ^°^C. Homogenates were serially diluted to certain concentrations and plated on GM17 plates at 30 ^°^C for 48 h. Bacterial colonies were counted and computed as CFU g^− 1^ of tissue.

#### 3.5.2 The anti-tumor mechanism of RH-FOLactis in pancreatic cancer

Following euthanasia via cervical dislocation, the tumor tissues and draining lymph nodes were isolated under aseptic conditions and immediately stored in pre-cooled physiological saline. Approximately 2 g of non-necrotic tumor tissue was minced and added to 3 mL of RPMI-1640 culture medium containing 1 mg/mL collagenase IV. The mixture was digested at 37 °C for 2 hours, then terminated with an equal volume of complete medium. The reaction was filtered through a 40 μm cell filter, centrifuged (1500 rpm, 5 min) and the supernatant was discarded. The cells were resuspended in 5 mL of red blood cell lysis buffer and centrifuged again, washed twice with PBS. The draining lymph nodes were ground, filtered and centrifuged, washed twice with PBS to obtain single-cell suspensions. Take the above single-cell suspension (approximately 1×10^6^ cells), add specific fluorescent antibodies (such as CD3, CD4, CD8, IFN-γ, TNF-α, CD11c, CD80, CD86,CD11b,Gr-1), incubate at 4°C in the dark for 30 minutes, centrifuge (1500 rpm, 5 minutes) and discard the supernatant. Wash twice with 1 mL PBS, and finally resuspend the cells with 200 μL PBS. Then, detect the proportions and activation status of immune cell subsets using a flow cytometer.

### 3.6 Statistical analysis

All statistical analyses were conducted using Graphpad Prism 9.5. T-tests and analysis of variance were used to compare differences among groups. Data were presented as mean ± standard error (Mean ± SEM), and p < 0.05 indicated statistical significance (where * indicated p < 0.05, ** indicated p < 0.01, and *** indicated p < 0.001).

## 4 Discussion

In this study, we systematically evaluated FOLactis-loaded P407 hydrogel (Gel-FOLactis), demonstrating its sustained-release properties and enhanced anti-tumor efficacy against pancreatic ductal adenocarcinoma (PDAC) through comprehensive characterization, cellular-level assays, and in vivo animal models. Furthermore, combining Gel-FOLactis with radiotherapy (RT) significantly increased the infiltration of DCs and cytotoxic T lymphocytes within the TME, yielding synergistic anti-tumor effects without detectable toxicity. Our findings establish that Gel-FOLactis alone or combined with RT improves therapeutic outcomes in “cold” pancreatic tumor murine models, highlighting the potential of hydrogel-based bacterial delivery systems for cancer immunotherapy and supporting the clinical translation of combined radio-immunotherapeutic strategies.

P407, an amphiphilic triblock copolymer composed of hydrophilic poly (ethylene oxide) and hydrophobic poly (propylene oxide) segments, self-assembles into micelles in aqueous media (31). The 20% P407 hydrogel exhibits sol-state fluidity at room temperature and undergoes rapid gelation at 37 °C, facilitating mucosal adhesion and localized drug retention (32). Gel-FOLactis demonstrated prolonged release kinetics, with sustained FOLactis release over 60 hours in vitro and 28 days in vivo. Beyond its controlled release capacity, P407 exhibits excellent biocompatibility and is widely employed in biomaterial and drug delivery applications (33). Consistent with prior reports, we confirmed that blank P407 hydrogel exerted no intrinsic anti-tumor effects on KPC cells, further validating its biosafety.

Previous studies indicated that FOLactis monotherapy promotes DCs maturation and cytotoxic T-cell infiltration, converting immunologically “cold” tumors into “hot” ones (20). Given that pancreatic cancer represents a “cold” tumor with limited response to conventional immunotherapy, we sought to enhance efficacy via localized sustained delivery (34). The significant tumor regression and successful reversal of the immunosuppressive TME achieved by the RH-FOLactis combination in this treatment-resistant model therefore represent a more substantial therapeutic advance. Moreover, and most critically, this study directly addresses a major translational limitation identified in the prior work—namely, the rapid clearance of free bacteria from the injection site.​ To this end, we engineered a P407 hydrogel-based sustained-release system (Gel-FOLactis) specifically to prolong local retention and provide continuous immune stimulation. The markedly superior anti-tumor efficacy and higher complete response rates observed with Gel-FOLactis, compared to free FOLactis, in our PDAC model provide direct experimental validation of this delivery strategy.​ This hydrogel platform is thus a pivotal innovation that moves the technology closer to clinical feasibility.

Radiotherapy remodels the TME by inducing immunogenic cell death and chemokine release, enabling T-cell recruitment along chemotactic gradients (35, 36). The combination of 8Gy RT with Gel-FOLactis significantly suppressed tumor growth. Mechanistic analyses revealed that combined therapy enhanced DCs activation in both TME and tumor-draining lymph nodes (TDLNs), subsequently amplifying CD8^+^T-cell-mediated tumor clearance. Antigen-specific T-cell responses were observed post-treatment, indicating robust activation of innate and adaptive immunity. The safety assessment, which included weight monitoring, histopathological analysis (H&E staining of major organs), and organ colony count, confirmed that the combination of Gel-FOLactis and RT performed well in terms of biological safety. This is consistent with the safety characteristics reported for FOLactis and P407 hydrogel (37, 38). Future studies will include cytokine profiling for comprehensive safety evaluation.

This study, conducted in a subcutaneous KPC allograft model, provides robust preliminary evidence for the anti-tumor efficacy and mechanism of RH-FOLactis. The KPC subcutaneous tumors also have a dense matrix, and the microenvironment is also inhibitory (39, 40). Our model preliminarily demonstrates the effectiveness of this method. Of course, there are indeed certain differences in the matrix and microenvironment between subcutaneous tumors and in situ tumors. Later, the effectiveness of the method needs to be verified in the in situ tumor model to promote its clinical application.

The therapeutic design of our approach, which combines the sustained local release of Flt3L and OX40L with radiotherapy-induced antigen release, is intended to initiate a systemic, tumor-specific T-cell response. This provides a mechanistic basis for the hypothesis that RH-FOLactis could induce abscopal effects, targeting non-irradiated metastases.​ To verify this hypothesis, a mature model of metastatic pancreatic ductal adenocarcinoma will be needed in the future. Additionally, future studies with larger animal numbers are warranted to conclusively establish the survival benefit of the combination regimen. Beyond efficacy validation, further mechanistic investigations are needed to advance clinical translation.

## 5 Conclusions

In summary, we developed a Gel-FOLactis vaccine demonstrating sustained release properties and potent anti-tumor effects in tumor-bearing mice. Furthermore, RH-FOLactis demonstrated a synergistic therapeutic effect, capable of activating dendritic cells (DCs) in the tumor microenvironment and enhancing the infiltration and function of cytotoxic T lymphocytes (CTLs), thereby triggering a powerful anti-tumor response. This combined strategy has high efficacy and good safety, demonstrating its strong potential for clinical translation.

## Data Availability

The raw data supporting the conclusions of this article will be made available by the authors, without undue reservation.

## Supplementary material

Supplementary Figure 1 Hematoxylin–eosin staining (H&E staining) of the major organs including the heart, liver, spleen, lungs, and kidneys in the KPC tumor model. The scale bar is 250μm.

Supplementary Figure 2 **(A, B)** The proportion of CD80^+^ and CD86^+^ in the TDLN group (n=6). **(C, D)** The proportion of CD80^+^ and CD86^+^ in tumor tissues (n=6).

Supplementary Figure 3 **(A)** *In vivo* bacterial distribution in major organs and blood at day 4 after treatment with 8Gy+Gel-FOLActis or 8Gy+FOLActis, shown as representative agar plates with serial dilutions. **(B)** Quantification of bacterial colony-forming units (CFU) per gram of tissue in tumors, TDLNs, and distant organs (heart, liver, spleen, lung, kidney, blood) for both treatment groups (mean ± SEM, n=3).

Supplementary Figure 4 T cells gating.

Supplementary Figure 5 DC cells gating.

Supplementary Figure 6 MDSC cells gating.

Supplementary Figure 7 Encapsulation in hydrogel does not compromise the ability of engineered bacteria to induce dendritic cell (DC) maturation. **(A)**. Representative flow cytometric dot plots showing the expression of CD80 and CD86 on bone marrow-derived dendritic cells (BMDCs) after 24-hour stimulation with conditioned media from different groups. **(B)** Quantification of the percentage of CD80+CD86+ mature DCs. Data are presented as mean ± SEM. ns, not significant (p > 0.05); ***p < 0.001. Groups include: Blank Gel (Gel), Free FOLactis (FOLactis), and Gel-Encapsulated FOLactis (Gel-FOLactis).

Supplementary Figure 8 8 Gy radiation induces immunogenic cell death (ICD) in tumor tissues. **(A)**. F low cytometric analysis of calreticulin (CRT) surface expression on tumor cells. **(B)** Quantification of the percentage of CRT-positive cells. Compared to the NS group, the 8 Gy radiation group (8Gy) showed significantly increased CRT surface exposure (*, p < 0.05). Data are mean ± SEM, n = 3.
