# Supplementary figures and images for: Radiotherapy-synergized in situ hydrogel vaccine with engineered Lactococcus lactis FOLactis potentiates anti-tumor immunity in pancreatic cancer
Source: Front Immunol. 2026 Jun 29;17:1789212. doi: 10.3389/fimmu.2026.1789212 (PMC13357422; doi:10.3389/fimmu.2026.1789212)

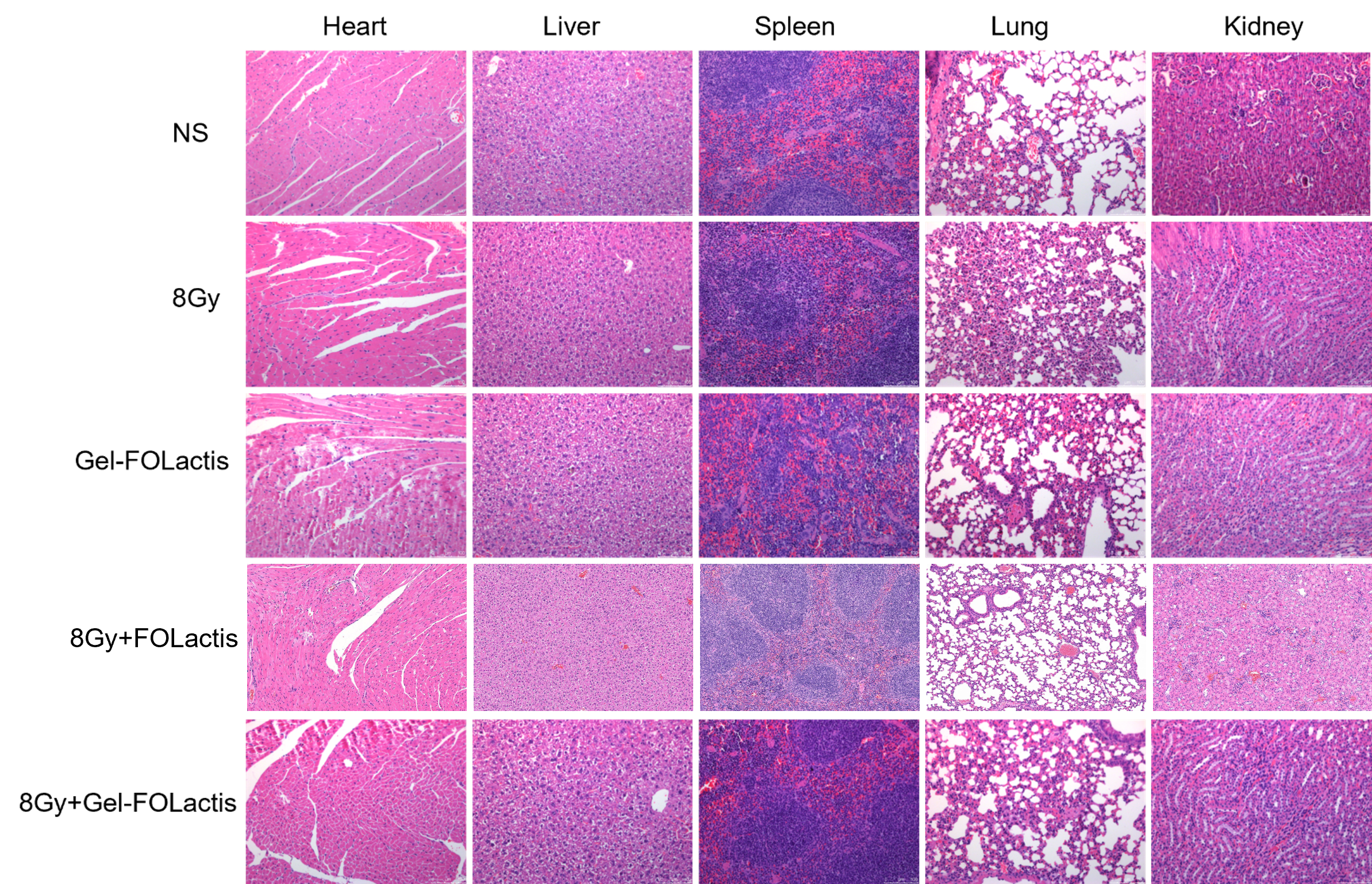

Supplement: Supplementary Figure 1 — Hematoxylin–eosin staining (H&E staining) of the major organs including the heart, liver, spleen, lungs, and kidneys in the KPC tumor model. The scale bar is 250μm. [file Image1.tif]

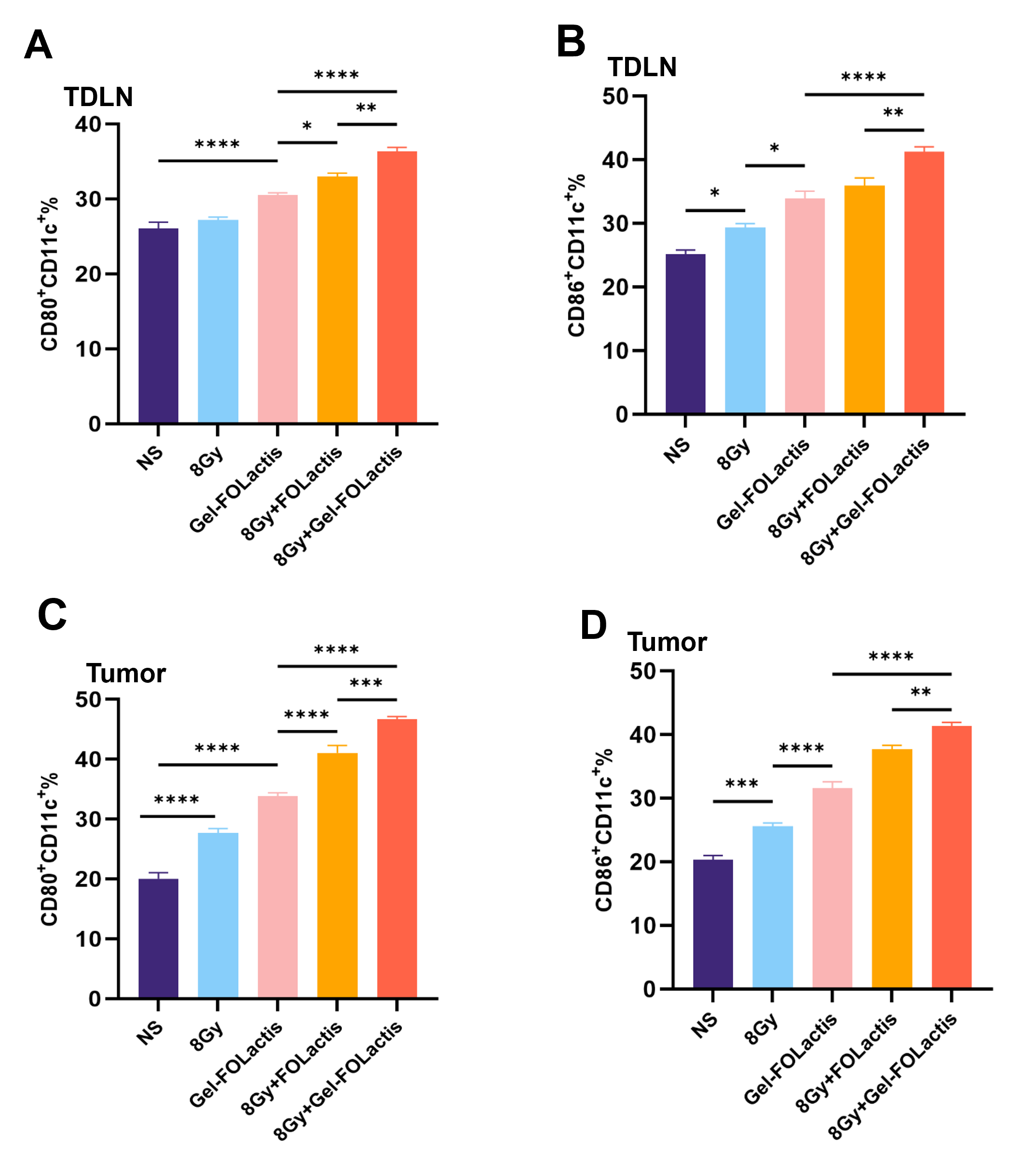

Supplement: Supplementary Figure 2 — (A, B) The proportion of CD80+ and CD86+ in the TDLN group (n=6). (C, D) The proportion of CD80+ and CD86+ in tumor tissues (n=6). [file Image2.tif]

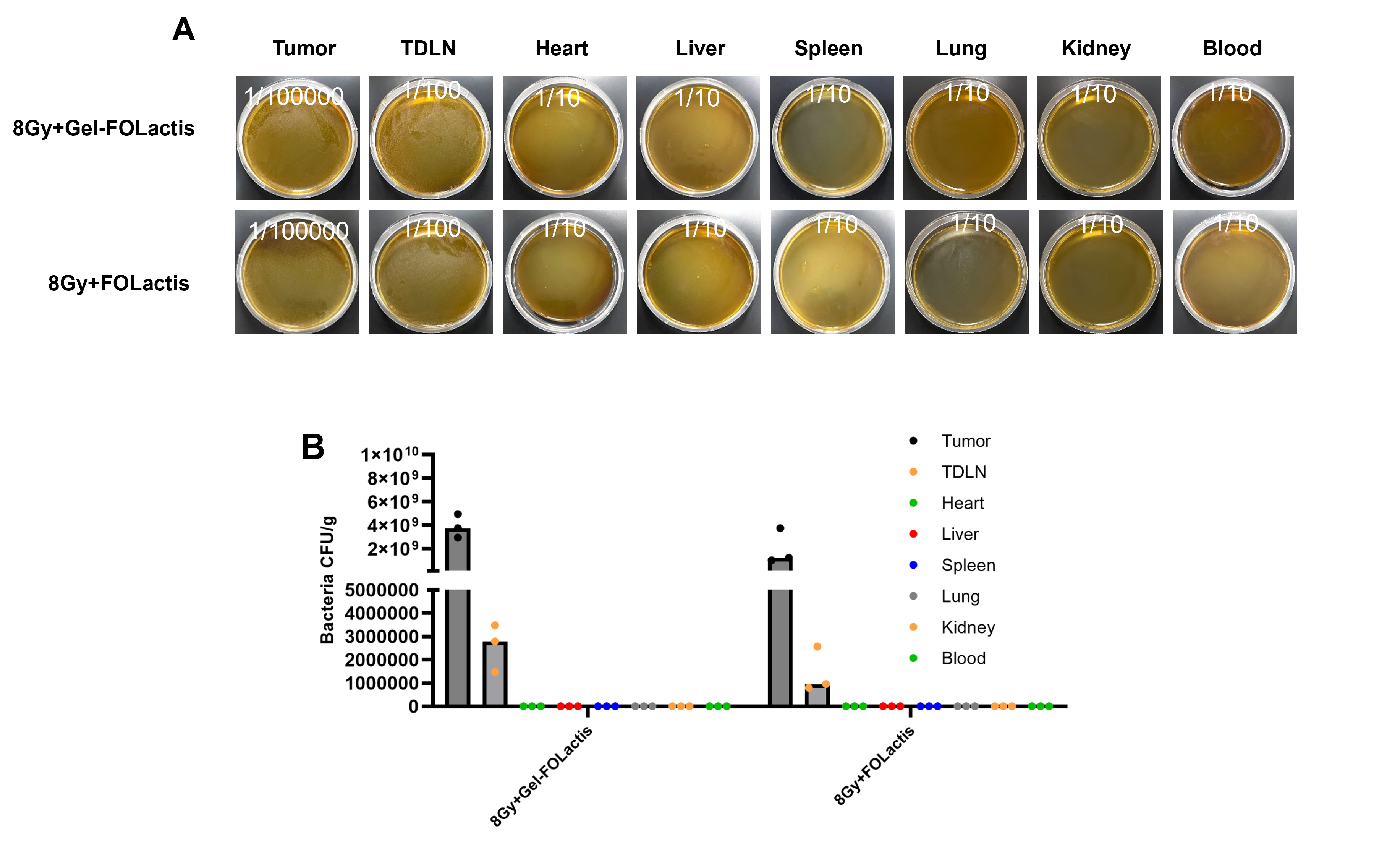

Supplement: Supplementary Figure 3 — (A) In vivo bacterial distribution in major organs and blood at day 4 after treatment with 8Gy+Gel-FOLActis or 8Gy+FOLActis, shown as representative agar plates with serial dilutions. (B) Quantification of bacterial colony-forming units (CFU) per gram of tissue in tumors, TDLNs, and distant organs (heart, liver, spleen, lung, kidney, blood) for both treatment groups (mean ± SEM, n=3). [file Image3.tif]

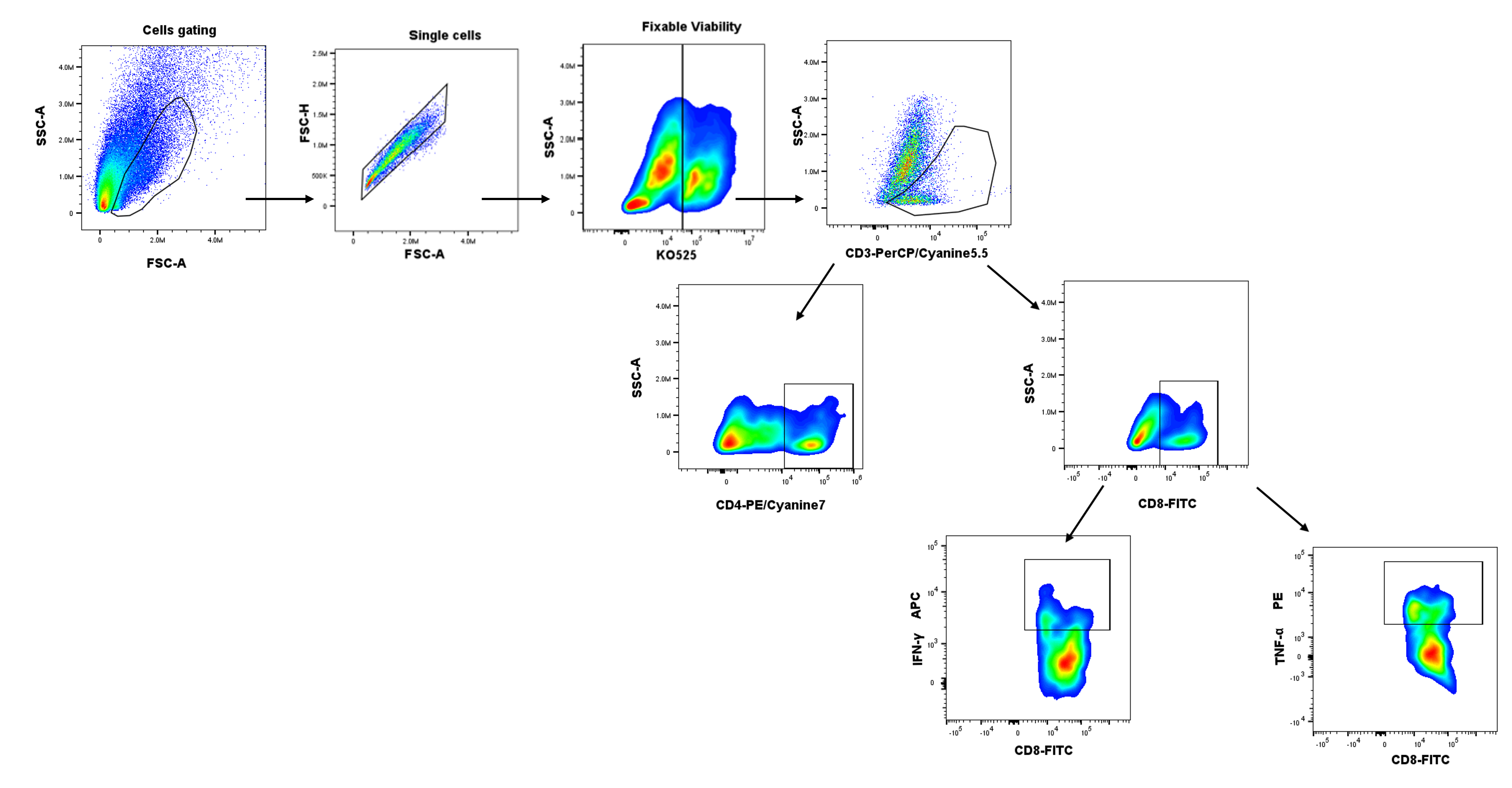

Supplement: Supplementary Figure 4 — T cells gating. [file Image4.tif]

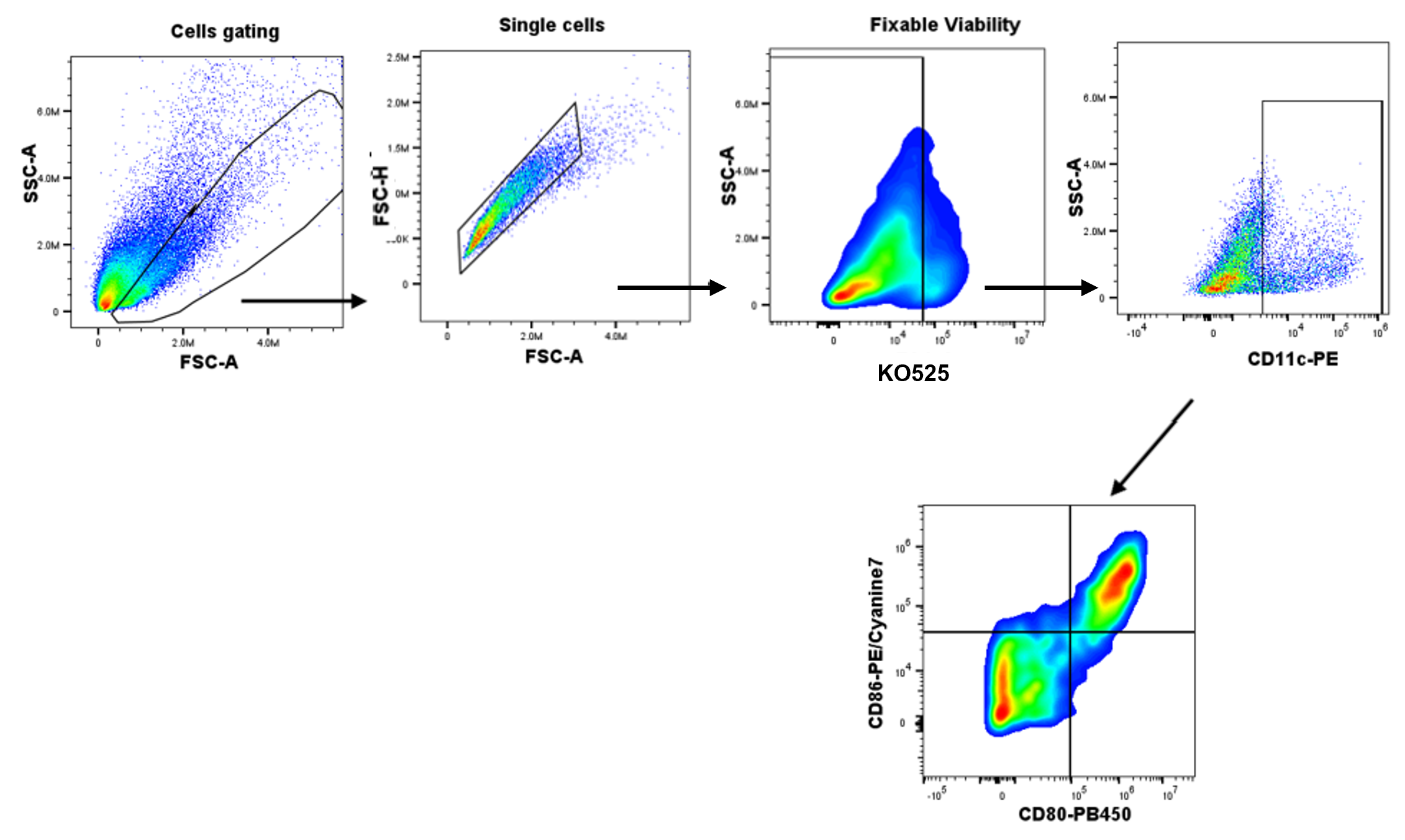

Supplement: Supplementary Figure 5 — DC cells gating. [file Image5.tif]

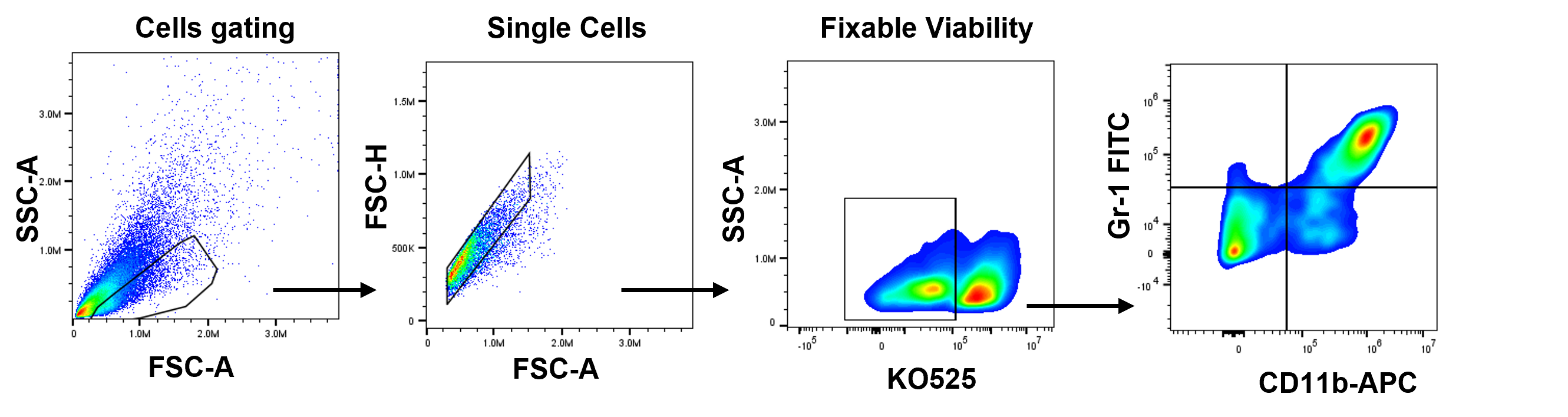

Supplement: Supplementary Figure 6 — MDSC cells gating. [file Image6.tif]

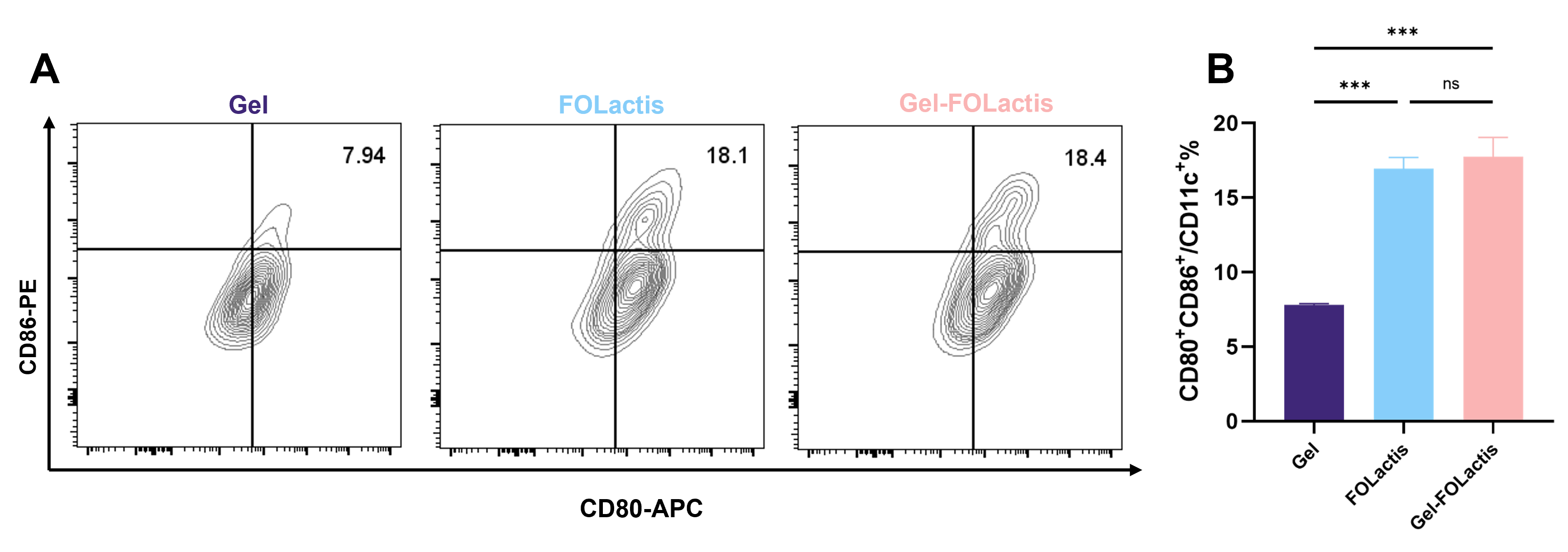

Supplement: Supplementary Figure 7 — Encapsulation in hydrogel does not compromise the ability of engineered bacteria to induce dendritic cell (DC) maturation. (A). Representative flow cytometric dot plots showing the expression of CD80 and CD86 on bone marrow-derived dendritic cells (BMDCs) after 24-hour stimulation with conditioned media from different groups. (B) Quantification of the percentage of CD80+CD86+ mature DCs. Data are presented as mean ± SEM. ns, not significant (p > 0.05); ***p < 0.001. Groups include: Blank Gel (Gel), Free FOLactis (FOLactis), and Gel-Encapsulated FOLactis (Gel-FOLactis). [file Image7.tif]

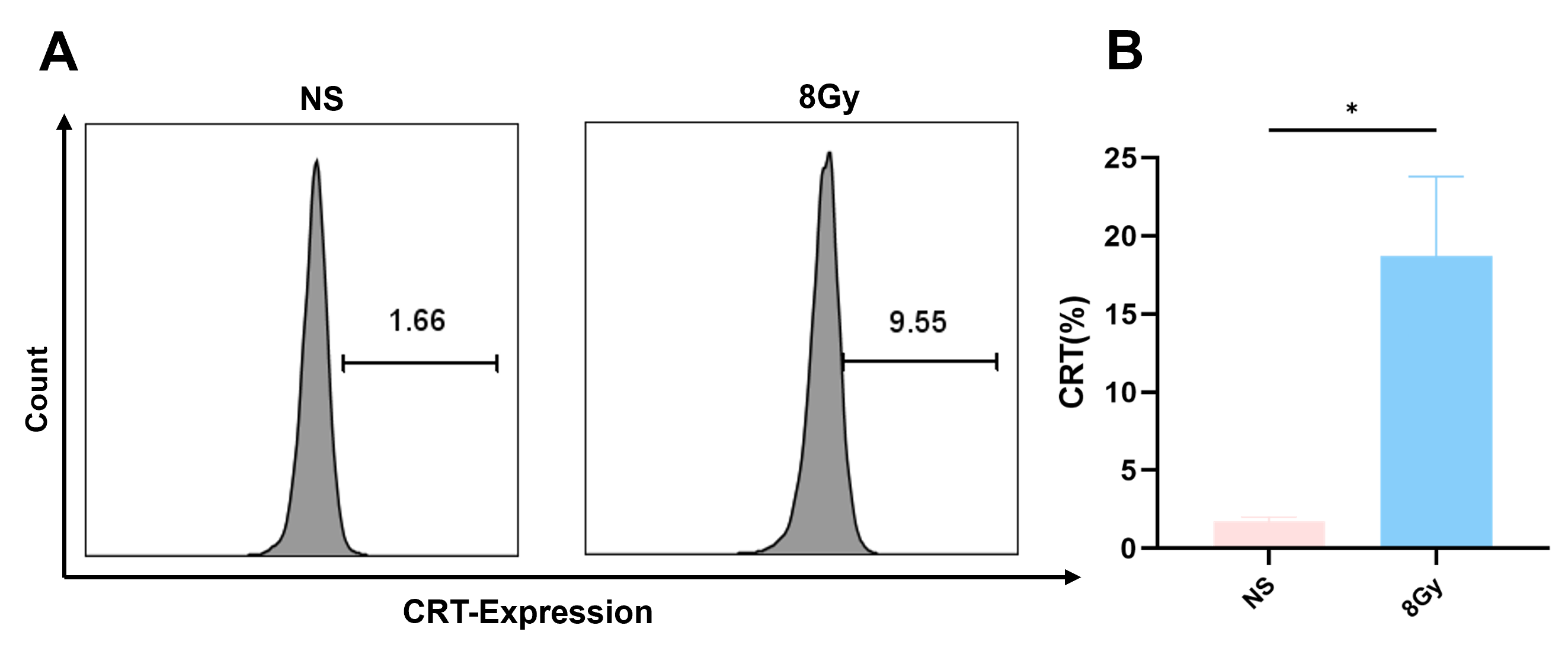

Supplement: Supplementary Figure 8 — 8 Gy radiation induces immunogenic cell death (ICD) in tumor tissues. (A). F low cytometric analysis of calreticulin (CRT) surface expression on tumor cells. (B) Quantification of the percentage of CRT-positive cells. Compared to the NS group, the 8 Gy radiation group (8Gy) showed significantly increased CRT surface exposure (*, p < 0.05). Data are mean ± SEM, n = 3. [file Image8.tif]
